# Competition and Symmetry in an Artificial Word Learning Task

**DOI:** 10.3389/fpsyg.2018.02176

**Published:** 2018-11-13

**Authors:** Brian Buccola, Isabelle Dautriche, Emmanuel Chemla

**Affiliations:** ^1^Laboratoire de Sciences Cognitives et Psycholinguistique (ENS, EHESS, CNRS), Département d'Études Cognitives, École Normale Supérieure, PSL Research University, Paris, France; ^2^Centre for Language Evolution, University of Edinburgh, Edinburgh, United Kingdom

**Keywords:** competition, symmetry, alternatives, psycholinguistics, semantics, pragmatics

## Abstract

Natural language involves competition. The sentences we choose to utter activate alternative sentences (those we chose not to utter), which hearers typically infer to be false. Hence, as a first approximation, the more alternatives a sentence activates, the more inferences it will trigger. But a closer look at the theory of competition shows that this is not quite true and that under specific circumstances, so-called *symmetric* alternatives cancel each other out. We present an artificial word learning experiment in which participants learn words that may enter into competition with one another. The results show that a mechanism of competition takes place, and that the subtle prediction that alternatives trigger inferences, and may stop triggering them after a point due to symmetry, is borne out. This study provides a minimal testing paradigm to reveal competition and some of its subtle characteristics in human languages and beyond.

## 1. Competition in language

### 1.1. First examples and description

In using language to communicate, the words and phrases that a speaker decides to use nearly always acquire an interpretation that goes beyond their strict, literal meaning. For example, if Alice utters to Bob the sentence in (1), then Bob might infer, among other things, that the animal Alice saw was not a cat or dog (or, at least, that Alice does not believe it was), but rather some more unusual animal like a raccoon, even though cats and dogs obviously count as animals, too.

(1) I saw an animal on my neighbor's porch this morning.

Similarly, if Alice utters to Bob the sentence in (2), then Bob will likely infer that Alice did not see both a dog *and* a cat on her neighbor's porch, even though, strictly speaking, seeing both animals counts as an instance of seeing one or the other.

(2) I saw a cat or a dog on my neighbor's porch this morning.

The process by which hearers draw these inferences has been the subject of much research and debate in semantic and pragmatic theory. However, starting with the pioneering work of Grice (1975), there is a consensus that, at its root, the process involves the hearer reasoning not just about what the speaker said, but also what the speaker could have said but chose *not* to say. That is, the things we say, as well as alternative things we could have said but chose not to, together affect the overall meanings of our utterances. In the case of (1), for example, if Alice had in fact seen a dog (and Alice knows she saw a dog), then it would be more appropriate for Alice to say so, even if (1) is true. Thus, if Alice chooses to utter (1) rather than the minimally different (3), in which *dog* replaces *animal*, then it is reasonable to infer that she did so because the animal she saw is *not* a dog (or any other option of the sort worth mentioning).

(3) I saw a dog on my neighbor's porch this morning.

In a parallel way, if Alice had seen both a cat and a dog (and Alice knows she saw both), then it would be more appropriate for Alice to say so, even if (2) is true. Thus, if Alice chooses to utter (2) rather than the minimally different (4), in which *and* replaces *or*, then it is reasonable to infer that she did so because she did *not* see both a cat *and* a dog.

(4) I saw a cat and a dog on my neighbor's porch this morning.

Grice (1975) coined the term *implicature* (and the associated verb *implicate*) to refer to the act of implying one thing by saying another. Thus, for instance, a speaker who utters (2) tends, we say, to implicate that (4) is false.

In sum, then, as speakers, the various things we can say when communicating a message “compete” with one another, so that what we choose to say and what we choose *not* to say together affect the final message we transmit.

### 1.2. Toward a theory of competition

As a first approximation toward a theory of competition in language, we might say that the use of an expression *φ* licenses the inference that *φ* was, in some sense, “better” or “more appropriate” than every alternative of *φ* that could have been used instead. We refer to this as the Competition Principle. (Our formulation in (5) is more general than the sorts of formulations found in the literature — e.g., Davis, 2014 and the references therein — and our reason for this is so that we may apply it to situations beyond traditional communicative settings.)

(5)**Competition Principle**. The use of *φ* implies that each alternative *ψ* of *φ* is less appropriate than *φ*.

This principle presupposes several notions that need to be spelled out: the notion of *use*, the notion of *appropriateness*, and the notion of an *alternative*.

In the context of the examples of above, and indeed in most of the relevant literature, to use an expression simply means to utter it, broadly speaking (i.e., to vocalize it, to sign it, to write it, and so on). In the context of our experimental task, this notion will take on a slightly broader meaning, which we will discuss later on.

The notion of appropriateness encompasses several possible things, because alternatives may be inappropriate (or less appropriate) for different reasons. For example, an alternative *ψ* of *φ* may be inappropriate simply because *ψ* is false (while *φ* is true), or *ψ* may be inappropriate because, although true, *ψ* is less informative, or specific, than *φ*. (This aspect of the Competition Principle is traditionally grounded in Grice's maxims of Quality and Quantity, respectively. We collapse them here for the sake of simplicity.)

Finally, the notion of alternative raises the question of what exactly “counts” as an alternative of *φ*. This is an important question that has received quite a bit of attention in the literature, the consensus being that alternatives need to be constrained in one way or another (for specific proposals, see, e.g., the *Horn scales* of Horn, 1972, and the theory of structurally defined alternatives of Katzir, 2007). We will not have much to add to this debate. For concreteness, we will adopt the simplistic view that the alternatives of *φ* are obtained by (recursively) replacing lexical elements in *φ* with other lexical elements from the given language. (For our experimental task, the choice of theory is immaterial, roughly because it will involve single-word expressions anyway.)

Putting everything together, we can say that, because of the Competition Principle, an utterance of (1) licenses the inference that the alternative in (3) is false (hence, that Alice saw an animal, but not a dog), and an utterance of (2) licenses the inference that the alternative in (4) is false (hence, that Alice saw a cat or a dog, but not both).

### 1.3. Symmetry

We have seen that alternatives create inferences. From the discussion so far, one may think that the more alternatives a sentence has, the more inferences one will draw from the use of that sentence. But this is not always so, because alternatives may cancel each other out, when a certain logical relation, known as *symmetry*, obtains between them (relative to the uttered sentence).

Abstractly first, symmetry arises when a sentence *φ* has two alternatives, *ψ*_1_ and *ψ*_2_, such that *ψ*_1_ and *ψ*_2_ can each be individually negated without contradicting *φ*, but their combined negation contradicts *φ*. In symbols, *φ*∧¬*ψ*_1_∧¬*ψ*_2_ is a contradiction, while *φ*∧¬*ψ*_1_ and *φ*∧¬*ψ*_2_ are not. In such cases, we say that *ψ*_1_ and *ψ*_2_ are symmetric alternatives (relative to *φ*), and that they create symmetry, because they cannot both be negated in a way that is compatible with *φ* — negating one forces the other to be true (Fox, 2007).

Concretely now, let *φ* be (2), and suppose that its two alternatives are (6a) (= *ψ*_1_) and (6b) (= *ψ*_2_) below. Then it is not possible for both (6a) to be false (Alice did not see a cat) and (6b) to be false (Alice did not see a dog), while at the same time the original sentence is true (Alice saw one or the other). So, disjunction (*φ* = *ψ*_1_∨*ψ*_2_) is a concrete case where two alternatives (*ψ*_1_ and *ψ*_2_) cannot both be negated, hence are symmetric.

(6)
  a. I saw a cat on my neighbor's porch this morning.
  b. b.I saw a dog on my neighbor's porch this morning.

In cases of symmetry, one might expect that in some contexts, *φ* could imply ¬*ψ*_1_ (rather than ¬*ψ*_2_), while in other contexts, *φ* could imply ¬*ψ*_2_ (rather than ¬*ψ*_1_). In actual fact, however, we observe that context cannot “break” symmetry (Fox and Katzir, 2011). Instead, hearers draw speaker uncertainty inferences regarding symmetric alternatives.

For example, (2), in addition to conveying that Alice did not see both a cat and a dog, also conveys that Alice is uncertain which of the two animals (a cat or a dog) she actually saw. How does the Competition Principle help us to understand this uncertainty inference? If Alice utters (2), and if (6a) and (6b) are alternatives of (2) (Sauerland, 2004), then the Competition Principle us that each of them was less appropriate than (2). However, by “less appropriate,” we cannot mean false, because it cannot be that (2) is true while (6a) and (6b) are both false (again, that would be a contradiction). So, it must mean something else. One natural possibility is that (2) is appropriate because Alice is certain that it is true, whereas each of (6a) and (6b) is less appropriate in virtue of Alice *not* being certain that it is true. If so, then this amounts to the observed uncertainty inference regarding the two symmetric alternatives (6a) and (6b).^1^

In short, more alternatives does not always equal more inferences. Sometimes, more alternatives introduces symmetry, which cancels out inferences that otherwise may have obtained (or converts them from plain negated inferences to uncertainty inferences).

### 1.4. Symmetry as a diagnosis of competition

In actual language use, symmetry does not seem to appear or disappear from context to context, but instead is rather stable across contexts. Abstractly, a more informative alternative *ψ* of *φ* either always has a symmetric partner (hence, the use of *φ* yields speaker uncertainty about *ψ*), or never does (hence, the use of *φ* yields the inference that *ψ* is false, provided the speaker is competent about *ψ*, and *ψ* is relevant). For example, when it comes to disjunction, as in (2), the conjunctive alternative, (4), never has a symmetric partner — this would be something like (7) below — so as a result, (2) invariably triggers the inference that (4) is false, rather than speaker uncertainty about (4) and (7).^2^ Conversely, a disjunction like (2) always has its individual disjuncts, (6a) and (6b), as alternatives, hence always exhibits symmetry, so as a result, (2) invariably triggers speaker uncertainty about (6a) and (6b), rather than the inference that one (or the other) of them is false.

(7) I saw a cat or a dog but not both on my neighbor's porch this morning.

A consequence of all this is that it can be relatively tricky to observe competition directly. If *φ* typically implies ¬*ψ*, then maybe this is simply because *φ* literally entails that *ψ* is false, or because *ψ* is extremely unlikely to begin with (given *φ*). For example, for (1), one might argue the inference that Alice did not see a dog is simply a contextual one (it's less likely for her to have seen a dog than, say, a raccoon — a weak argument, admittedly). Conversely, for (2), one might argue that *or* is inherently exclusive, i.e., that *φ* *or* *ψ* literally means “*φ* or *ψ* but not both”.

In a similar fashion, if *φ* typically implies speaker uncertainty about *ψ*_1_ and *ψ*_2_, then maybe this is simply because *φ* literally entails such uncertainty. For example, perhaps the literal meaning of *or* encodes something about the knowledge state of the speaker who uses it, so that it actually entails speaker uncertainty about the individual disjuncts.

In short, because competition is difficult to observe directly, one may wonder whether there is any competition going on in these cases to begin with. Of course, linguists have developed intricate diagnostics to argue that these *are* examples of competition, e.g., embedding them in downward-entailing (roughly, negative) contexts and observing that the relevant inferences disappear. For example, *I did not see a cat or a dog on my neighbor's porch this morning* does not trigger any speaker uncertainty inferences, nor does it convey the denial of speaker uncertainty about the individual disjuncts (if *or* literally encoded speaker uncertainty, then this sentence could mean “it is not the case that I saw a cat or a dog but I don't know which,” which would be true in a scenario where Alice saw a cat or a dog and Alice knew which — an impossible reading of the sentence).

Nevertheless, our goal here is to explore whether there is a way to observe the Competition Principle more directly. We propose to do so using symmetry as the diagnosis for the presence of competition, by manipulating the presence or absence of symmetry across experimental contexts (something that does not readily happen in everyday linguistic contexts). Specifically, we report on an artificial word learning experiment which had the following goal: to see whether we could create competition between two nonce words — a word *w* that applies to more than one kind of object, and a more specific/informative word *w*_1_ that applies to a strict subset of what *w* applies to — and observe its effect, and then to remove that effect by introducing a third word, *w*_2_, such that *w*_1_ and *w*_2_ are symmetric relative to *w*.

Our artificial word learning experiment involved tasks in which communicative cooperativeness (hence, traditional Gricean maxims) seemed to play little or no role (there was no speaker-hearer, for instance). Capitalizing on this aspect, a secondary goal of ours was to see whether a general, i.e., not specifically conversational, notion of competition — something like our Competition Principle in (5) — could be detected, especially since it is often assumed in the Gricean literature that Gricean principles are grounded in more general principles of rationality.^3^ Up to now, this idea has never been tested. Our results suggest a positive answer: the Competition Principle *does* play a role in non-conversational tasks like the ones we used.

## 2. Experiment

The Competition Principle seems to be at the heart of pragmatic enrichment during communication in natural language, but it can often be difficult to assess exactly what is in competition, what role symmetry plays, etc. We present an experimental study that investigates whether we may observe the Competition Principle somewhat more directly over the course of acquisition of nonce words, by manipulating the presence or absence of alternatives and symmetry across experimental contexts.

### 2.1. Task summary and hypothesis

**Figure 1:**
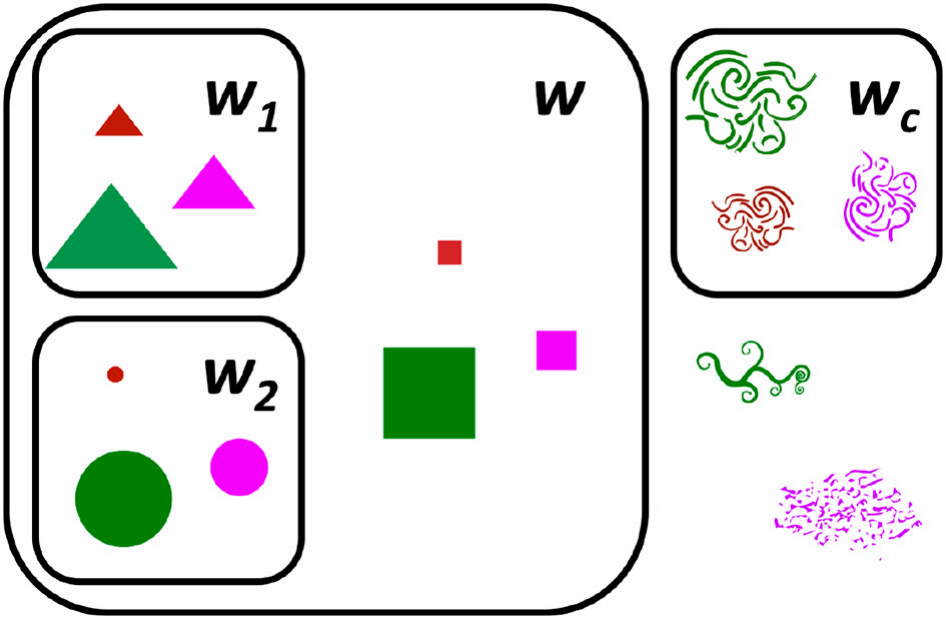
Participants learned four novel words: three critical words (*w*, *w*_1_, and *w*_2_) and one control word (*w*_*c*_). *w* applied to (at least) two kinds of objects (e.g., both triangles and circles), while *w*_1_ applied to just one of the two kinds (e.g., triangles), and *w*_2_ applied to just the other of the two kinds (e.g., circles).

The goal of the task was to learn three new words — *w*, *w*_1_, and *w*_2_ — where *w* applied to (at least) two kinds of objects (e.g., both triangles and circles), while *w*_1_ applied to just one of the two kinds (e.g., triangles), and *w*_2_ applied to just the other of the two kinds (e.g., circles) (see Figure 1).

**Figure 2:**
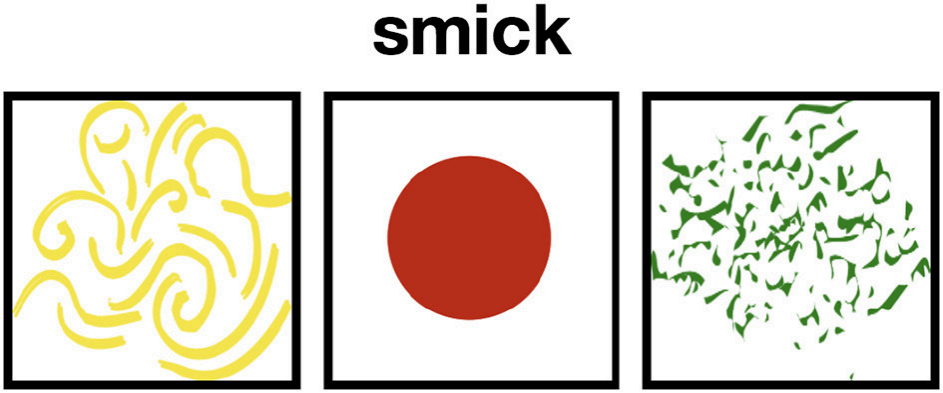
Example of a trial.

To learn the meaning of words, participants observed a series of displays containing one of the words to be learned and a collection of objects with different properties (see Figure 2). They then picked an object from the collection and received feedback.

**Figure 3:**
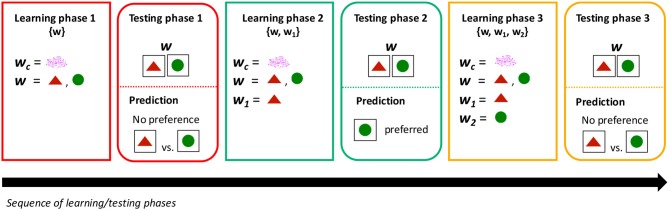
Experimental design, conditions, and predictions. Participants were administered a sequence of learning and testing phases. The Competition group was administered the 3 learning phases (followed by their testing phases), as they learned *w*, *w*_1_, and *w*_2_ sequentially. The No-competition group skipped the second learning phase ({*w, w*_1_}), as *w*_1_ and *w*_2_ were learned simultaneously. The testing phase, following each learning phase, consisted of the same critical trials: participants were presented with the word *w* and both *w*_1_-type-objects and *w*_2_-type-objects. We predicted that after learning *w* only, there should be no preference in choosing between *w*_1_-type-objects vs. *w*_2_-type-objects. Critically, we predicted that after introducing a single alternative (after learning *w* and *w*_1_ but not *w*_2_), *w*_1_-type-objects should be selected *less* than before, due to competition between *w* and *w*_1_. Finally, introducing a second alternative (or learning both alternatives simultaneously in the case of the No-competition group) should remove the effect of competition; thus, we expect no preference between *w*_1_-type-objects and *w*_2_-type-objects.

We tested participants' understanding of *w* when presented with both *w*_1_-type-objects and *w*_2_-type-objects at different learning stages: after they learned *w* only, after they learned *w* and *w*_1_ but not *w*_2_, and after they learned all three words *w*, *w*_1_, and *w*_2_ (see Figure 3). The idea then was to gradually introduce alternatives: first a unique alternative, which may trigger inferences through the Competition Principle, and then yet another alternative that may create symmetry, and could therefore remove the inferential effect of competition. More specifically, our hypothesis was the following: after learning *w*, but before learning *w*_1_ or *w*_2_, participants should choose indiscriminately between the two kinds of objects (or perhaps with some measurable bias); after learning *w* and *w*_1_, participants should choose *w*_2_-type-objects more so than before, due to competition between *w* and *w*_1_; and finally after learning *w*, *w*_1_, and *w*_2_, participants should go back to choosing indiscriminately, due to symmetry between *w*_1_ and *w*_2_.

### 2.2. Method

All data and scripts for their analysis are available at https://semanticsarchive.net/Archive/DJmNjYxY/.

#### 2.2.1. Ethics statement

This study was carried out in accordance with the recommendations of the Comité d'Éthique de la Recherche en Santé (2013/46). The protocol was approved by the Comité d'Éthique de la Recherche en Santé (2013/46). In accordance with the Declaration of Helsinki, prior to participating in this online study, all participants were presented with the informed consent document and instructions stating that by clicking “I accept” they indicated their consent to participate in the study.

#### 2.2.2. Participants

Fifty-three adults were recruited through Amazon's Mechanical Turk (25 females; *M* = 38 years; all native speakers of English) and compensated $1.80 for their participation. Participants were randomly assigned to one of two groups (see Design below): the Competition group (*N* = 26) and the No-competition group (*N* = 27). One additional participant was excluded in the Competition group for failing to pass the learning criteria.

#### 2.2.3. Procedure

Participants were tested online. They were instructed that their task was to learn new words by associating them with objects displayed on the screen. In the instructions, participants were given a screenshot of a trial involving a word (not used during the test) and a set of objects. No information about the number of to-be-learned words was given. For each trial, a word was displayed, first alone for 500 ms to attract participants' attention to the word, then together with a collection of 3 objects, aligned horizontally, below the word (see Figure 2). Participants were asked to click on the object they believed to be associated with the word. The experiment consisted of several learning and testing phases (see Design below).

During the learning phases, participants received feedback on their response after each trial. The feedback was displayed in a horizontal bar positioned at the top of the screen. The bar turned green and displayed the prompt “Correct!” for correct responses, and turned red and displayed the prompt “Incorrect” for incorrect responses. Correct responses had 2 s of feedback before the next trial, while incorrect responses had 6 s of feedback to increase attention to the task.

During the testing phases, participants did not receive any feedback: once they responded, the experiment continued with the next trial. Each testing phase was preceded by a warning to participants (“You will not receive feedback for the next couple of events.”) displayed for 4 s in the same top horizontal bar used for the feedback.

Participants' answers as well as their response times were recorded on each trial. At the end of the experiment, there was a final questionnaire asking participants about their age, native language, and country.

#### 2.2.4. Stimuli

The space of objects included 3 geometric shapes (circles, triangles, and squares) and 3 organic shapes (clouds of dots, clouds of curly lines, and spiraling branches). For variability, objects also varied across two irrelevant dimensions: colors (red, yellow, blue, green, and pink) and size (small, medium, and big), leading to 15 possible configurations per object.

We chose 4 novel words from a list of pseudowords obeying the rules of English phonotactics (*blicket, dax, diti, smick, tupa, fep, bosa, moop, zud, vash*, and *gaddle*).

#### 2.2.5. Design

Each participant learned four words over the course of the experiment: *w*, *w*_1_, *w*_2_, and *w*_*c*_. *w* applied to the 3 geometrical shapes (i.e., circles, triangles, and squares), whereas *w*_1_ applied to just one (e.g., triangles), and *w*_2_ to another one (e.g., circles) (see Figure 1). *w*_*c*_ was a control word that applied to one of the 3 organic shapes to encourage participants to pay attention to the words and not click systematically on any of the geometrical shapes present in the display. The target objects associated with *w*_1_, *w*_2_, and *w*_*c*_ were randomized across participants.

The experiment was divided into several learning phases, each followed by a testing phase. We used a between-subject design in which some subjects received three learning and testing phases (the Competition group), and others two (the No-competition group). In the former case, participants first learned *w* and *w*_*c*_, then *w*_1_, and finally *w*_2_; in the latter case, participants first learned *w* and *w*_*c*_, then *w*_1_ and *w*_2_ simultaneously (see Figure 3 for a graphical illustration of the time course of the experiment).

#### 2.2.6. Learning phases

All trials featured a single target object with two randomly chosen distractors such that there was only a single correct response. Trials were presented in blocks to control for the amount of learning received for each word. Details describing the exact number of trials per word per block in each learning phase can be found in the Supplemental Material. Participants were exposed to a minimum of 3 blocks. The learning phase ended when participants responded correctly for all trials in a block. If they answered more than 250 trials without reaching the learning criteria, the experiment continued normally but we discarded their responses (*N* = 1).

#### 2.2.7. Testing phases

The testing phases always consisted of 4 critical trials interspaced with the same type of trials seen during the previous learning phase (3 trials per word learned until that point; see the Supplemental Material for a precise description). In the critical trials, participants were presented with *w*, together with a collection of objects that contained both a *w*_1_-type-object (e.g., a triangle) and a *w*_2_-type-object (e.g., a circle). These critical trials were placed at the beginning of the testing phases, and interspaced by one other trial.

#### 2.2.8. Conditions and predictions

There were three conditions that depended on the training a participant received. In the {*w*} condition (no alternative), participants had learned *w* but not *w*_1_ or *w*_2_; in the {*w, w*_1_} condition (one alternative), participants had learned both *w* and *w*_1_ but not *w*_2_; and in the {*w, w*_1_, *w*_2_} condition (two alternatives, symmetric), participants had learned *w*, *w*_1_, and *w*_2_.

The testing phase, with the same critical trials, was administered after each of these different learning phases, allowing us to test the effect of symmetry in participants' lexicon on their responses on the critical trials. We measured the proportion of *w*_1_-type-objects vs. *w*_2_-type-objects that participants picked when presented with the word *w* and both kinds of objects. The critical trials and the predictions associated with each condition are illustrated in Figure 3. Our predictions were the following: in the {*w*} condition (after learning *w*, but before learning *w*_1_ or *w*_2_), participants should choose indiscriminately between the two kinds (or perhaps with some measurable bias); in the {*w, w*_1_} condition (after learning *w* and *w*_1_, but before learning *w*_2_), participants should choose *w*_2_-type-objects more so than before, due to competition between *w* and *w*_1_; and in the {*w, w*_1_, *w*_2_} condition (after learning *w*, *w*_1_, and *w*_2_), participants should go back to the same response rate observed in the {*w*} condition, due to symmetry between *w*_1_ and *w*_2_. Critically, in the No-competition group, who are not learning *w*_1_ and *w*_2_ sequentially but simultaneously (and thus do not receive the {*w, w*_1_} condition), there should be no difference in their response rate between the {*w*} and the {*w, w*_1_, *w*_2_} conditions, since both *w*_1_ and *w*_2_ immediately compete with *w*, and the effects of competition are thus canceled out due to symmetry.

#### 2.2.9. Data analysis

The data analysis was conducted using the lme4 package (Bates et al., 2015) of R. In a mixed logit regression (Jaeger, 2008), we modeled the selection of *w*_1_-type-objects (coded as 0 or 1) compared to *w*_2_-type-objects during the critical test trials. All responses on critical trials were included in the analysis since participants always picked either *w*_1_- or *w*_2_-type-objects, and never a distractor object. The model included two categorical predictors with their interaction: Group (Competition vs. No-Competition) and Condition ({*w*} vs. {*w, w*_1_} vs. {*w, w*_1_, *w*_2_}) as well as a random intercept and random slopes for Condition for participants. The resulting R syntax for the model was: *w*_1_-type-objects-selection ~ Condition * Group + (1 + Condition | Participant).

### 2.3. Results

**Figure 4:**
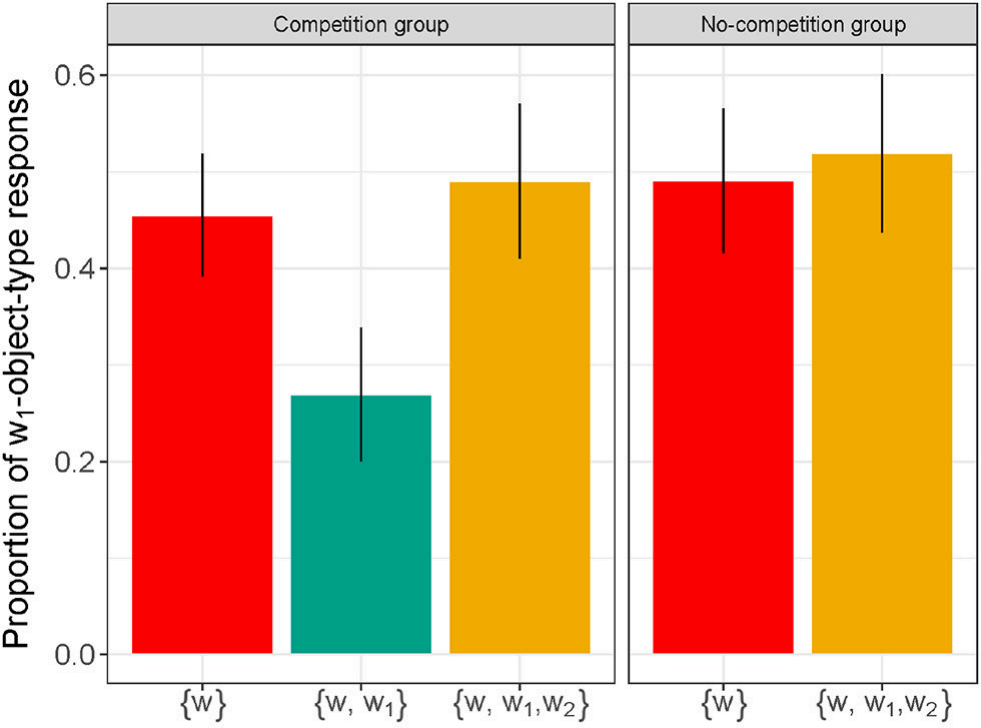
Proportion of *w*_1_-type-object responses obtained during the critical trials for each condition (relevant words learned were {*w*} vs. {*w, w*_1_} vs. {*w, w*_1_, *w*_2_}) and for each group (Competition vs. No-Competition). Error bars indicate standard errors of the mean.

Figure 4 reports the average proportion of *w*_1_-type-object responses during the critical trials by condition ({*w*} vs. {*w, w*_1_} vs. {*w, w*_1_, *w*_2_}) and group (Competition vs. No-Competition).

The outputs of the models are in the Supplemental Material, with the full script available at https://semanticsarchive.net/Archive/DJmNjYxY/Competition_analysis.R. Over the conditions present in the two groups ({*w*} and {*w, w*_1_, *w*_2_}), there was no main effect of Group (χ^2^ = 0.27; *p* = 0.87), nor a significant interaction between Condition and Group (χ^2^ = 0.01; *p* = 0.91), illustrating that both groups responded in the same way in the {*w*} condition (*M*_*comp*_ = 0.46; *SE*_*comp*_ = 0.06 vs. *M*_*no*−*comp*_ = 0.49; *SE*_*no*−*comp*_ = 0.07) and in the {*w, w*_1_, *w*_2_} condition (*M*_*comp*_ = 0.49; *SE*_*comp*_ = 0.08 vs. *M*_*no*−*comp*_ = 0.52; *SE*_*no*−*comp*_ = 0.08).

Critically, there was a main effect of Condition (χ^2^ = 13.61; *p* < 0.01). Participants' responses were sensitive to the presence or the absence of symmetry in their lexicon: participants in the Competition group selected less *w*_1_-type-objects in the {*w, w*_1_} (*M* = 0.27; *SE* = 0.07) condition than in the surrounding {*w*} (β = 1.72; *z* = 2.66; *p* < 0.01) and {*w, w*_1_, *w*_2_} (β = 1.82; *z* = 2.86; *p* < 0.01) conditions. In other words, learning *w*_1_ created visible effects of competition, and further learning its symmetric alternative, *w*_2_, removed these effects.

### 2.4. Discussion

Participants were sensitive to the presence/absence of alternatives and symmetry in their lexicon: when asked to pick an object corresponding to a word *w*, participants preferred to pick a *w*-compatible object for which there was no alternative word that also applied, i.e., to pick a *w*_2_-type-object (for which there was no alternative word yet) rather than a *w*_1_-type-object (for which there was an alternative word, *w*_1_). This competition effect between the referents of the word *w* — those that were *w*_1_-compatible and those that were not — was removed when participants learned another alternative word, *w*_2_, that applied to just the other kind of objects labeled by *w*, due to symmetry between *w*_1_ and *w*_2_.

Our task involved nonce words that have translation equivalents in the English lexicon (e.g., *shape, triangle, circle*). Can our result be explained by participants' existing lexicon? We believe it is unlikely. If participants used their existing lexicon in the task, then we would expect no competition in the {*w, w*_1_} condition, as the English lexicon would still be symmetric in this case. Therefore, the presence of a competition effect, and its subsequent removal after introducing *w*_2_, suggest that participants use only their newly acquired lexicon in the task.

Another possible alternative explanation for the effect is that participants answered strategically with the goal of balancing out their *w*_1_-type (e.g., triangle) and *w*_2_-type (e.g., circle) responses. As a result, when they had a choice between triangle and circle, if they had responded triangles often enough, they may have decided to pick the circle. When learning an alternative word during a learning phase, participants were given opportunities to respond with the shape corresponding to that word, and so in the following testing phase, they may have thus seized opportunities to give the other options. This explanation predicts that the symmetry effect should be mitigated by this behavior, since the third learning phase does not completely erase the imbalance between the two alternatives (triangles have been selected more often than circles across all learning phases). Yet this is not what we observe. Also, although it is phrased differently, this description may actually be just another version of the Competition Principle: the reason why participants want to balance their triangle and circle responses, all things being equal, may very well be because of a competition effect (selecting triangles repetitively when prompted with the alternative word, *w*_1_, would encourage participants to pick circles over triangles when prompted with a compatible word, *w*). All in all, however, after debriefing a few people who did the tasks in our lab, it seems that the direct competition explanation is a better match for explaining our participants' behavior.

Finally, an anonymous colleague (p.c.) notes that in testing phase 3, perhaps participants construe *w* as referring to the third, *w*_3_-type-object (via competition between *w* and both *w*_1_ and *w*_2_), and are at chance only because the *w*_3_-type-object is not an available option on the critical test trials, not because symmetry is at play, as we claim. To spell this idea out a bit more explicitly, once *w*_1_ and *w*_2_ are both learned, then in the critical trial, if competition were at play, then participants would construe *w* as “*w* but not *w*_1_ and not *w*_2_,” i.e., as *w*_3_; but since *w*_3_ is not an available option, the overall result is a kind of “contextual contradiction.” As such, competition leads to a crash, and so competition evidently must not be at play (is “turned off”), and so participants choose randomly between *w*_1_ and *w*_2_, just like in phase 1. If this is correct, then one could still present this situation as a case of symmetry blocking inferences: *w*_1_ and *w*_2_ are symmetric relative to *w* and the *context* of the trial (which excludes *w*_3_ as an option), and that is why participants do not invariably go for just one or the other. Put differently, *w*_1_ and *w*_2_ are still symmetric relative to *w* *in the context of the trial*, in the sense that “*w* and not *w*_1_ and not *w*_2_” is a contextual contradiction given the absence of any *w*_3_-type-object. (In other cases of symmetry, “*w* and not *w*_1_ and not *w*_2_” would be a plain contradiction, as discussed in §1.3 for the case of disjunction, *φ* = *ψ*_1_∨*ψ*_2_.) So, here, contextual symmetry blocks inferences, just as in other cases of symmetry.

In sum, our results suggest that the Competition Principle may be observed directly during an artificial word learning task as a function of the absence or presence of symmetry at different learning stages of an artificial lexicon.

## 3. General discussion and conclusion

Our results suggest that competition (with and without symmetry) arises spontaneously in artificial word learning tasks, even though the experimental context is not a traditional conversational exchange in any obvious sense. This in turn means that participants appear to apply something like the Competition Principle during the task. Specifically, they presumably apply a kind of reasoning like the following:

- {*w*} condition: No competition. Choose freely between the *w*_1_-type-object and the *w*_2_-type-object.
- {*w, w*_1_} condition: The trial uses *w*, but it could have used *w*_1_ instead. Therefore, *w*_1_ might have been less appropriate. Thus, the *w*_1_-type-object might be less appropriate than the *w*_2_-type-object. Choose the *w*_2_-type-object.
- {*w, w*_1_, *w*_2_} condition: The trial uses *w*, but it could have used *w*_1_ or *w*_2_. Therefore, *w*_1_ and *w*_2_ might have each been less appropriate. But it would not follow that the *w*_1_-type-object is less appropriate than the *w*_2_-type-object, or vice versa. Thus, neither is more or less appropriate than the other. Choose freely between them.

### 3.1. The minimal ingredients for competition

It is worth stressing that our experimental task involves the absolute minimal ingredients required for observing competition in all of its intricacy, including the role played by symmetry (there are just three words: *w*, *w*_1_, and *w*_2_). That these ingredients turn out to also be sufficient is remarkable, particularly in an experimental context that bears little resemblance to everyday conversational contexts (there is no speaker-hearer, for example). Our results therefore suggest that, when even the minimal ingredients for competition are present, humans instinctively and spontaneously employ something like the Competition Principle.

### 3.2. Beyond human reasoning

Non-human animals, such as monkeys, dogs, and birds, are capable of learning words, and they are also capable of applying strategic reasoning in various tasks. It has even been suggested that some monkeys apply a kind of Competition Principle in their natural alarm call system (Schlenker et al., 2014, 2016). A natural question is whether we can directly detect the Competition Principle at play in non-human animal behavior. Our experimental design is sufficiently simple that it should be straightforward to examine this question, something we hope to do in future work.

## Author contributions

All authors contributed to most aspects of the project, including designing the experiment. BB wrote the first draft of the manuscript. ID programmed the experiment and performed the statistical analysis. EC initiated the project.

### Conflict of interest statement

The authors declare that the research was conducted in the absence of any commercial or financial relationships that could be construed as a potential conflict of interest.

## Footnotes

^1^Sauerland (2004) (building on Gazdar, 1979; Soames, 1982; Horn, 1989) provides a more formal implementation of this reasoning process.

^2^The question of why (4) but not (7) is an alternative of (2), and how the theory of alternatives should explain this fact, is an instance of the so-called *symmetry problem* (Fox, 2007; Katzir, 2007; Fox and Katzir, 2011), which does not concern us here.

^3^The idea that conversation is a cooperative enterprise grounded in rational behavior originates with Grice himself (Grice, 1975) (for discussion, see also Levinson, 1983). Recent game-theoretic approaches to this idea include Franke (2011) and Bergen et al. (2016).
