# Supplementary material for: Competition and Symmetry in an Artificial Word Learning Task
Source: Front Psychol. 2018 Nov 13;9:2176. doi: 10.3389/fpsyg.2018.02176 (PMC6282061; doi:10.3389/fpsyg.2018.02176)
Supplement: Supplementary file 1 [file Data_Sheet_1.pdf]

Supplemental material

Learning phases. The experiment consisted of 2 or 3 learning phases depending on the experimental group. Trials were presented in blocks to control for the amount of exposure for each word. Below we detail the type and the number of trials per block for each learning phase corresponding to the condition:

- **$\{w\}$ (common to both the Competition and the No-competition group)**: Participants learned w and the control word w_c. A block of trials consisted of 6 trials: 3 for w and 3 for w_c. The 3 trials for w (dubbed $w-1$, $w-2$ and $w-3$; see Table 1) featured a different target object each (i.e. circle, triangle, square) to ensure that participants would associate w with each of the geometrical shapes an equal number of times. Participants took on average 3.75 blocks (23 trials) to complete this phase.
- **$\{w, w_1\}$ (Competition group only)**: Participants learned w_1. A block of trials consisted of 18 trials: 12 trials for w_1, and 3 trials each for w and w_c. The trials for w were presented to ensure that participants did not forget what they had learned in the previous learning phase. The 12 trials for w_1 were of 3 possible types (w_1-1, $w_1-1,2$ and $w_1-1,3$; see Table 1). Participants took on average 4.15 blocks (75 trials) to complete this phase.
- **$\{w, w_1, w_2\}$ (Competition group only)**: Participants learned w_2. A block of trials consisted of 21 trials: 12 trials for w_2, and 3 trials each for w, w_1, and w_c. The 12 trials for w_2 were of 3 possible types (w_2-2, $w_2-2,1$ and $w_2-2,3$; see Table 1). Participants took on average 3.65 blocks (77 trials) to complete this phase.
- **$\{w, w_1, w_2\}$ (No-competition group only)**: Participants learned w_1 and w_2 simultaneously. A block of trials consisted of 30 trials: 12 trials each for w_1 and w_2, and 3 trials each for w and w_c. The 12 trials for w_1 and w_2 were of the same type as the ones seen by the Competition group in the $\{w, w_1\}$ and $\{w, w_1, w_2\}$ learning phases. Participants took on average 4.26 blocks (128 trials) to complete this phase.

Testing phases. A testing phase was administrated to participants after each learning phase. Each testing phase was composed of 4 critical trials (see Method section or alternatively Table 1) plus 3 trials (one per trial type) for each test word seen during the previous learning phase. This resulted in a total of 7 trials for the testing phase of condition $\{w\}$, 10 trials for the condition $\{w, w_1\}$, and 13 trials for the condition $\{w, w_1, w_2\}$.

On the non-critical test trials, participants' average accuracy ranged between 98% and 100% across each learning phase and experimental group, suggesting that they accurately learned the meaning of the words taught during the previous learning phase.

Statistical model. The model was declared rank deficient because one group did not have the second condition, and after dropping the relevant parameter it yielded the outputs given in Table 2 and Table 3.

**Trial type**	**Description**
$w-1$	w_1-target + 2 organic shapes
$w-2$	w_2-target + 2 organic shapes
$w-3$	remaining geometric shape + 2 organic shapes
$w-1,2$ (critical test)	w_1-target + w_2-target + 1 organic shape
w_1-1	w_1-target + 2 organic shapes
$w_1-1,2$	w_1-target + w_2-target + 1 organic shape
$w_1-1,3$	w_1-target + remaining geometric shape + 1 organic shape
w_2-2	w_2-target + 2 organic shapes
$w_2-2,1$	w_2-target + w_1-target + 1 organic shape
$w_2-2,3$	w_2-target + remaining geometric shape + 1 organic shape

Table 1: Trial types used during the course of the experiment. The format of the trial type name follows the template: target word - list of subscripts of the critical objects presented on screen.

	Estimate	Std. Error	z value	p-value
Intercept (condition=test2,group=Competition)	−1.9659	0.7531	−2.611	0.00904 **
condition=test1	1.7229	0.6486	2.656	0.00790 **
condition=test3	1.8221	0.6367	2.862	0.00421 **
group=No-competition group	0.3720	0.9815	0.379	0.70469
condition=test1:group=No-competition	−0.1005	0.8875	−0.113	0.90981

Table 2: Fixed effects.

	(Intr)	Condition=test1	Condition=test3	Group=No-comp
Condition=test1	−0.856			
Condition=test3	−0.525	0.542		
Group=No-comp	−0.428	0.307	−0.250	
Condition=test1,Group=No-comp	0.249	−0.345	0.327	−0.832

Table 3: Correlation of fixed effects.
